# Eyeblink Classical Conditioning in Alcoholism and Fetal Alcohol Spectrum Disorders

**DOI:** 10.3389/fpsyt.2015.00155

**Published:** 2015-11-02

**Authors:** Dominic T. Cheng, Sandra W. Jacobson, Joseph L. Jacobson, Christopher D. Molteno, Mark E. Stanton, John E. Desmond

**Affiliations:** ^1^Department of Neurology, Johns Hopkins University School of Medicine, Baltimore, MD, USA; ^2^Department of Psychiatry and Behavioral Neurosciences, Wayne State University School of Medicine, Detroit, MI, USA; ^3^Department of Psychiatry and Mental Health, University of Cape Town, Cape Town, South Africa; ^4^Department of Human Biology, University of Cape Town, Cape Town, South Africa; ^5^Department of Psychology, University of Delaware, Newark, DE, USA

**Keywords:** alcoholism, ethanol, cerebellum, fetal alcohol spectrum disorders, eyeblink classical conditioning, associative learning

## Abstract

Alcoholism is a debilitating disorder that can take a significant toll on health and professional and personal relationships. Excessive alcohol consumption can have a serious impact on both drinkers and developing fetuses, leading to long-term learning impairments. Decades of research in laboratory animals and humans have demonstrated the value of eyeblink classical conditioning (EBC) as a well-characterized model system to study the neural mechanisms underlying associative learning. Behavioral EBC studies in adults with alcohol use disorders and in children with fetal alcohol spectrum disorders report a clear learning deficit in these two patient populations, suggesting alcohol-related damage to the cerebellum and associated structures. Insight into the neural mechanisms underlying these learning impairments has largely stemmed from laboratory animal studies. In this mini-review, we present and discuss exemplary animal findings and data from patient and neuroimaging studies. An improved understanding of the neural mechanisms underlying learning deficits in EBC related to alcoholism and prenatal alcohol exposure has the potential to advance the diagnoses, treatment, and prevention of these and other pediatric and adult disorders.

## Introduction

Alcohol is one of the most widely abused substances in the world (1) and can have a major impact on health and professional and personal relationships. One reason for this negative societal impact is that excessive alcohol consumption often leads to long-term learning and memory impairments. In this mini-review, we will outline exemplary animal and human findings that guide our current understanding of how chronic alcohol exposure alters neural structure and function underlying a fundamental form of learning, eyeblink classical conditioning (EBC). Specifically, this mini-review will focus on alcohol use disorders (AUD) in adults and fetal alcohol spectrum disorders (FASD) in children.

**Figure 1:**
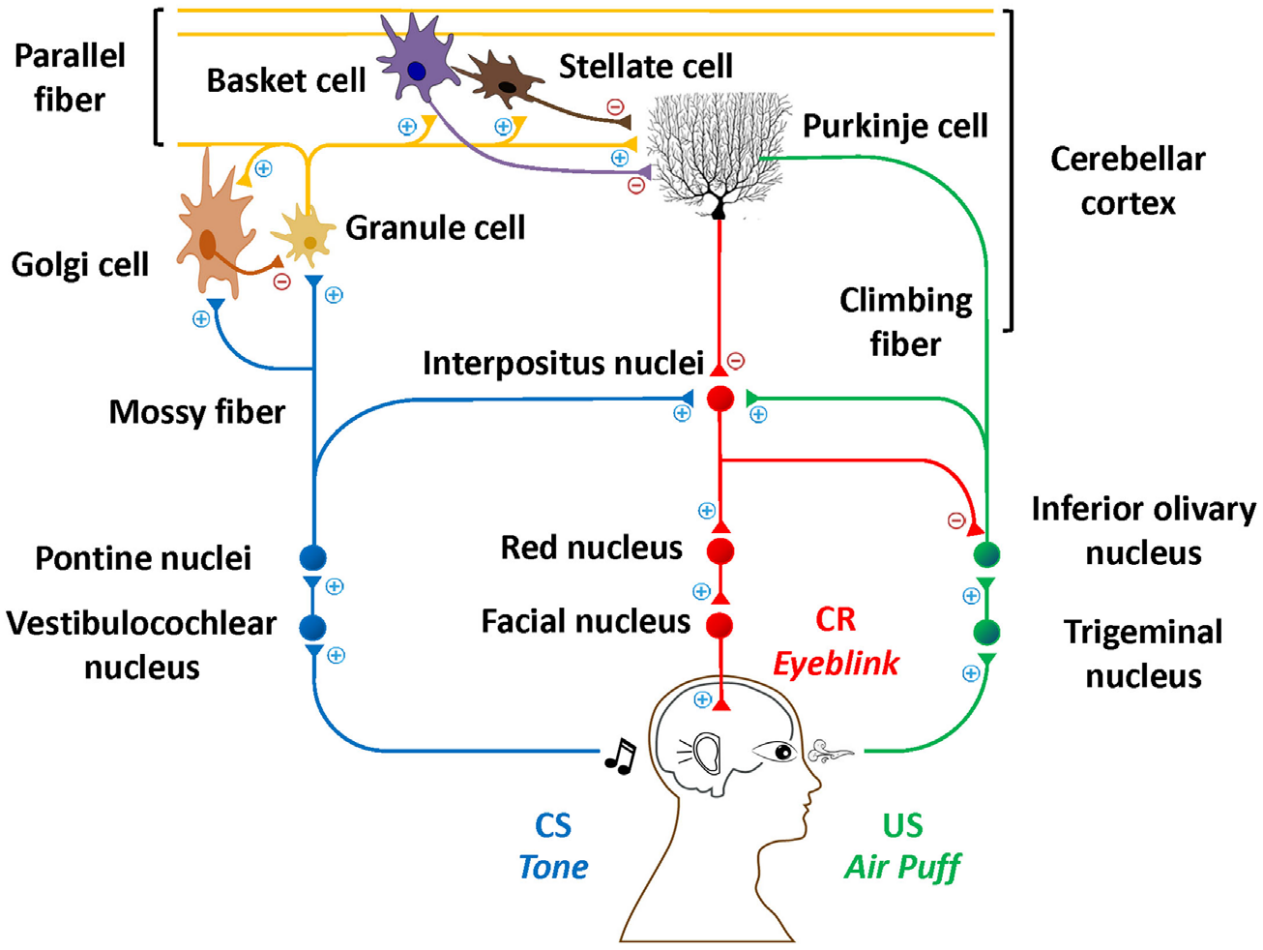
**Essential neural circuitry of eyeblink conditioning**. Blue lines indicate the conditioned stimulus pathway. Green lines indicate the unconditioned stimulus pathway. Red lines indicate the conditioned response pathway. Excitatory and inhibitory synapses are represented by + and −, respectively.

One area of the brain that is targeted in AUD and FASD is the cerebellum (2, 3). Although excessive alcohol consumption affects many other brain regions (4–6), this mini-review will focus on the cerebellum due to its critical involvement in EBC (7) and the particular vulnerability of the cerebellum to alcohol exposure (8, 9). This line of research has produced overwhelming evidence that the cerebellum and associated structures are critically important for EBC. Specifically, contributions from the cerebellar cortex, particularly in lateral lobule VI (10, 11), and cerebellar deep nuclei (12, 13) have been documented in both animals and humans. Figure 1 depicts this well-documented circuitry.

Eyeblink classical conditioning involves the pairing of a neutral conditioned stimulus (CS; e.g., a tone) and an unconditioned stimulus (US; e.g., a corneal airpuff). The US is often a biologically salient stimulus sufficient to elicit an unconditioned response (UR; e.g., a blink). Following multiple CS–US pairings, an organism learns to produce a conditioned response (CR) in anticipation of the US presentation, suggesting that an association between the CS and US has been learned. EBC is a simple, yet elegant model of learning, which can already be assessed in humans by 5 months of age (14) and represents a foundation on which more complex learning is built (15, 16). Understanding the etiology of fundamental learning impairments that accompany alcohol-related disorders may have potential to foster new approaches to early diagnoses, intervention, and effective treatments and presents a model for studying effects of other pediatric and adult disorders as well as effects of other drugs or environmental contaminants.

## Laboratory Animal Work

### Structural Alterations (Mature Cerebellum)

There is extensive laboratory animal evidence showing that chronic intake of alcohol is associated with neuroanatomical changes in the cerebellum (17). A common observation is shrinkage of the cerebellum. In the adult rat, these volumetric reductions may be due to death and atrophy of cells in the Purkinje, granular, and molecular layers of the cerebellar cortex (18–21). In addition to degenerative changes in cell bodies, morphological changes to dendrites and axons have also been reported (22–24). Combined treatments of thiamine deficiency and alcohol exposure have led to axon terminal degeneration in the deep cerebellar nuclei, the sole output region for the cerebellum (25). Fewer synapses between parallel fibers and Purkinje cells (26) and a significant decrease in the number of dendritic microtubules have been found in alcohol-fed adult rats (27). At the molecular and cellular level, γ-aminobutyric acid_A_ (GABA_A_) is altered by chronic alcohol consumption (28), whereas there is an overexpression of glutamate and a prolonged opening of mitochondrial permeability in the cerebellum following alcohol withdrawal (29).

### Structural Alterations (Developing Cerebellum)

Cerebellar structural abnormalities also appear in the developing cerebellum as a result of excessive early alcohol exposure. This damaging effect appears to be sensitive to time of alcohol exposure as rats receiving alcohol on postnatal day 4 suffered up to 50% Purkinje cell loss, whereas later exposure (postnatal days 8/9) resulted in less severe (15%) cell loss (30, 31). Alcohol-related damage in granule cells has also been investigated and cell vulnerability again appears to be greatest early in development (postnatal days 4/5) (32, 33). The structural integrity of the cerebellar deep nuclei, a region believed to be crucially important for EBC memory formation and storage (7), has been shown to be susceptible to chronic alcohol consumption. Binge-like and moderate neonatal exposure to alcohol was sufficient to produce behavioral deficits in EBC associated with significant deep nuclear cell loss in adult rats (34, 35). During development, even a single exposure to alcohol introduced subcutaneously was sufficient to promote cellular apopotosis in the deep cerebellar nuclei (36).

### Functional Differences (Mature Cerebellum)

Abnormal cerebellar functioning is another consequence of chronic alcohol exposure. Very little attention has been given to the chronic effects of alcohol on the cerebellum in adult laboratory animals. To the best of our knowledge, only one study to date has examined these effects. In mature mice, chronic alcohol consumption resulted in a decrease in simple and complex spike firing and an increase in complex spike duration and pause in Purkinje cells but no differences were detected in Golgi cell firing patterns (37).

### Functional Differences (Developing Cerebellum)

Most of our current knowledge on the functional consequences of chronic alcohol exposure stems from work on the developing cerebellum. Following alcohol exposure during pregnancy, *in vitro* experiments using a long-term depression (LTD) induction protocol showed parallel fiber long-term potentiation (LTP) in cerebellar slices in alcohol-exposed juvenile mice but LTD in control mice (38). Furthermore, *in vivo* experiments showed that simple spike firing rates in Purkinje cells increased and showed faster oscillations of local field potentials in exposed mice relative to controls (38). These exposed mice also exhibited impaired EBC, further supporting the hypothesis that cerebellar LTD in Purkinje cells is crucial for the timing of eyeblink CRs (39). Interestingly, other *in vitro* electrophysiology experiments showed that alcohol exposure led to relatively greater inhibitory inputs to the Purkinje cells in the vermis (40). In the cerebellar deep nuclei, activity in the interpositus nucleus of the cerebellum was diminished and did not develop as rapidly in neonatal alcohol-exposed rats relative to controls during EBC (41, 42).

### Learning Deficits

Since the cerebellum is vulnerable to chronic alcohol exposure and this structure plays a critical role in EBC, prolonged alcohol use is likely to result in learning deficits. Surprisingly, to date, there are no laboratory animal eyeblink conditioning studies investigating the role of chronic alcohol consumption in adulthood.

By contrast, there have been several animal studies on effects of pre- and neonatal exposure. Neonatal rats exposed to alcohol during the equivalent of the human third trimester showed learning deficits in standard delay EBC (43) as well as more complex EBC protocols, including trace conditioning, discrimination, and reversal learning (44, 45). The effects of alcohol on EBC also appear to be dose dependent, with higher dosages producing greater impairments (45, 46). Binge-like and even moderate exposure to alcohol during development produces EBC deficits that persist into adulthood, suggesting long-lasting permanent cerebellar damage (35, 47). This evidence is consistent with studies that report a significant correlation between learning and the number of deep cerebellar nuclear cells in alcohol-exposed rats (34). Finally, interventions to ameliorate neonatal alcohol-related learning deficits have been met with mixed results. MK-801 administration, choline supplementation, and a combination of exercise and environmental enrichment mitigate behavioral EBC deficits, suggesting neuroprotective or other ameliorative effects (48–50), whereas vitamin E did not reduce alcohol-related EBC deficits (51).

## Human Work

### Structural Alterations (Mature Cerebellum)

Consistent with laboratory animal findings, human data also indicate that chronic alcohol consumption has harmful effects on the structural integrity of the adult cerebellum (4, 52). Structural MRI has revealed gray matter reductions in the cerebellar hemispheres and vermis in AUDs (53). Furthermore, cerebellar gray matter volume loss was correlated with poor neuropsychological performance and early age of first drinking (54). Diffusion tensor imaging (DTI) showed that recovered AUDs had diminished white matter fibers relative to healthy controls, suggesting that impaired connectivity may partially mediate some of these behavioral deficits (55). Human histological studies report significant Purkinje cell loss in the cerebellar hemispheres and vermis as a result of years of alcohol abuse (9, 56, 57).

### Structural Alterations (Developing Cerebellum)

As indicated above, animal models predict that alcohol exposure damages the developing cerebellum. These findings are also consistent with human studies: autopsy reports of children prenatally exposed to large quantities of alcohol describe malformations in the cerebellum characterized by reduced size and disorganization (58). In addition, cerebellar dysgenesis was reported in 10 of 16 FAS autopsies (59). Modern neuroimaging data agree with these observations, as exposed children had proportionately greater reductions in cerebellar cranial vault and volume (60, 61), including a 15% reduction in cerebellar volume in children with FAS (8). Specifically, significantly smaller cerebellar hemispheres and vermis were found in exposed relative to healthy children (62, 63). Differences in white matter integrity [lower fractional anisotropy (FA) and greater perpendicular diffusivity] between alcohol-exposed and non-exposed children have been identified in the middle cerebellar peduncles, fibers shown to be important in animal models of EBC (64, 65). Children with FAS also showed lower FA bilaterally in the superior peduncles. Finally, using *in vivo* (1) H magnetic resonance spectroscopy (MRS) to examine neurochemical differences in the cerebellar deep nuclei, Du Plessis et al. (66) found that prenatal alcohol exposure was associated with lower levels *N*-Acetylaspartate (NAA) and glycerophosphocholine + phosphocholine (Cho) and higher levels of glutamate plus glutamine (Glx).

### Functional Differences (Mature and Developing Cerebellum)

Consistent with these structural findings, evidence from functional magnetic resonance imaging (fMRI) studies suggests fMRI brain activations are also affected by alcoholism. In a finger tapping task, AUD subjects tended to exhibit more extensive and bilateral cerebellar activation than healthy controls (67). Greater right superior cerebellar activity during a Sternberg working memory task was assessed in AUD subjects (68). In an auditory language task, AUD subjects showed greater fMRI activations in the cerebellar vermis, despite comparable behavioral performance to healthy controls (69). Children diagnosed with fetal alcohol syndrome (FAS) or partial FAS (PFAS) showed greater cerebellar activation in a working memory n-back task relative to healthy children (70). Rhythmic tapping elicited greater activation in children with FASD in crus I and vermis IV–V (71). This pattern of greater activation by adults and children may represent compensatory mechanisms during each task.

### Learning Deficits

Similar to laboratory animals, humans also show alcohol-related deficits in EBC. Impaired standard delay eyeblink conditioning (CS and US co-terminate) was seen in amnesic Korsakoff patients and recovered, uncomplicated AUDs (72). These findings were extended to more complex conditioning protocols. During temporal discrimination, in which two distinct CSs with two different interstimulus intervals (ISI) were presented, AUDs’ peak CR latency at the long ISI was significantly shorter relative to healthy controls, demonstrating a deficit in adaptive CR timing (73). Trace conditioning is a procedure that incorporates a stimulus free period between offset of the CS and onset of the US. Naive AUDs showed learning deficits in trace conditioning, whereas AUDs previously trained in delay conditioning showed comparable trace conditioning to naive control subjects (74). AUDs who were successful at learning a delay discrimination protocol (i.e., learn that one CS predicts the US, whereas another CS predicts its absence) were impaired when the contingencies were reversed, suggesting an inability to learn new adaptive associations (75).

Similar to adults, children with FASD demonstrate remarkably consistent conditioning deficits. In a cross-sectional study comparing children with FASD, attention deficit hyperactive disorder (ADHD), dyslexia, and healthy controls, the children with FASD and dyslexia showed conditioning impairments relative to the healthy children and different patterns than those seen in children with ADHD (76). In the first prospective longitudinal study on EBC in children with FASD, Jacobson et al. (77) extended these findings by presenting additional trials (up to 150 trials) to 5-year-old children diagnosed with FAS, PFAS, heavily exposed non-syndromal (HE) children, and controls. Despite the additional training opportunity, none of the children with FAS met criterion for conditioning, whereas 75% of the controls did (77). In another cohort of school-aged children, 66.7% of the children with FAS failed to meet criterion on the delay task, and only 16.7% of the FAS and 21.4% of HE group met criterion for trace conditioning in comparison to 66.7% of healthy controls (78). Odds ratio data from a logistic regression analysis showed that the children with FAS were 7.7 times more likely to fail to meet criterion on the delay task compared with controls and 10.0 times more likely on the trace conditioning task. Similarly, the HE group was 5.1 times more likely to fail to meet criterion on delay and 7.3 times more likely on trace. In both the 5-year and school-age studies, IQ did not differentiate the children who reached criterion on delay and trace EBC from those who failed, indicating that it could not be a mediator of the effect of fetal alcohol exposure on performance on either EBC task; nor was ADHD responsible for the observed alcohol-related pattern of EBC impairment seen in the two cohorts. Collectively, these findings strongly support the view that prenatal alcohol exposure has deleterious effects on children’s ability to demonstrate successful EBC and thus has the potential to serve as a biobehavioral marker of prenatal alcohol impairment as well as a useful tool to assess the efficacy of an intervention (79).

## Discussion

The damaging effects of alcoholism on the cerebellum and EBC have been well-documented in animal and human investigations. This mini-review summarizes some exemplary laboratory animal and human studies (see Table 1). Chronic, excessive alcohol consumption leads to neuroanatomical alterations in the adult and/or fetal cerebellum, including neuronal loss and white matter degradation. Alcohol exposure also triggers abnormal cerebellar activity as shown through electrophysiology and neuroimaging methodologies. The combination of these effects likely underlies the conditioning deficits seen by these two populations.

**Table 1:** **Effects of alcohol on cerebellar structure, function, and eyeblink conditioning reported in the literature**.

Animals			Humans	
	Reference	Comments	Reference	Comments
Structural alterations	(32)	Purkinje and granule cell loss (D)	(9)	Purkinje cell volume loss (M)
	(36)	Purkinje and deep cerebellar nuclear cell loss (D)	(8)	Cerebellar volume loss (D)
	(30)	Purkinje cell loss (lobules I–V, IX, and X) (D)	(63)	Hypoplasia of cerebellar vermis (D)
	(34, 35)	Deep cerebellar nuclear cell loss (D)	(54)	Cerebellar gray matter loss correlated with neuropsych. tests (M)
	(33)	Purkinje and granule cell loss (postnatal days 4–5) (D)	(55)	Diminished white matter fiber (M)
	(21)	Purkinje and granule cell loss (M)	(59)	Cerebellar dysgenesis in 10 of 16 FAS autopsies (D)
	(27)	Dendritic microtubules loss (M)	(58)	Cerebellar reduction and disorganization (D)
	(24)	Longer terminal dendritic segments in Purkinje cells (M)	(66)	Differences in cerebellar neurochemisiry (D)
	(25)	Deep cerebellar nuclear axon terminal degeneration (M)	(65)	Cerebellar peduncles damage (D)
	(18)	Granule cell loss (M)	(60, 61)	Reductions in cerebellar cranial vault and volume (D)
	(19, 22)	Longer and reduced Purkirje dendritic spines (M)	(57)	Cell loss in cerebellar vermis (M)
	(23)	Increased climbing fibers (M)	(62)	Cerebellar vermis volume reduction (D)
	(20)	Purkinje and granule cell loss (M)	(64)	Cerebellar peduncles damage (D)
	(26)	Fewer synapses between parallel fibers and Purkinje cells (M)	(53)	Cerebellar vermis gray matter deficits (M)
	(31)	Purkinje cell loss (postnatal days 4–5) (D)	(56)	Reduced Purkinje cell density in the vermis (M)
Functional differences	(41)	No single-unit activity changes in cerebellar deep nuclei (D)	(69)	Greater fMRI activity in cerebellar vermis (M)
	(40)	Greater inhibitory inputs to Purkinje cells (D)	(68)	Greater fMRI responses in lobule VI (M)
	(42)	Slower increases in deep nuclear activity (D)	(70)	Greater cerebellar fMRI activation (D)
	(37)	Purkinje cell firing differences (M)	(71)	Greater crus I and vermis IV–V activation (D)
	(38)	Purkinje cell firing differences (D)	(67)	More extensive cerebellar fMRI activation (M)
Learning deficits	(44)	Impaired EBC discrimination learning (D)	(76)	Impaired delay EBC (D)
	(34, 35, 47)	Impaired delay EBC (D)	(75)	Impaired EBC discrimination and reversal learning (M)
	(45)	Impaired trace EBC (D)	(77, 78)	Impaired delay and trace EBC (D)
	(43)	Impaired delay EBC (D)	(74)	Impaired trace EBC (M)
			(72, 73)	Impaired delay and temporal EBC discrimination (M)

*A summary of animal and human work investigating how excessive alcohol consumption affects the cerebellum and eyeblink conditioning. M and D indicate effects on the mature and developing cerebellum, respectively*.

One limitation in this field of study is that alcohol affects multiple regions of the brain outside the cerebellum. Affected and connected areas may exert influences on cerebellar structures, making results difficult to interpret. Future work should consider the cerebellum as part of a larger network. This fundamental associative learning task is clinically relevant because it represents a foundation on which more complex learning is built. Studies of environmental exposures, such as alcohol, on EBC have the potential to provide new information about the EBC neural circuitry and behavioral performance and to elucidate vulnerable neural structures that are uniquely recruited during basic learning processes. A comparison of EBC and neuroimaging findings between adults with AUD and children with FASD to determine common neuroanatomical targets of alcohol abuse is an important goal. Moreover, EBC has the potential to identify impairment related to different exposures and in different pediatric and adult disorders, such as ADHD, schizophrenia, FASD, and AUD. This work could lead to assessment of degree of behavioral and cerebellar impairment in AUD and aid in early identification of fetal alcohol-affected children as well as assessment of efficacy of new interventions and treatments. Future interventions could involve the use of neuromodulatory tools, such as transcranial magnetic stimulation and transcranial direct current stimulation, as a way to alter brain activation in an effort to improve learning in AUD and FASD individuals. Finally, this learning model could also be used to identify at-risk individuals, thereby leading to effective prevention strategies.

## Conflict of Interest Statement

The authors declare that the research was conducted in the absence of any commercial or financial relationships that could be construed as a potential conflict of interest.
